# Direct Angiotensin AT2 Receptor Stimulation Using a Novel AT2 Receptor Agonist, Compound 21, Evokes Neuroprotection in Conscious Hypertensive Rats

**DOI:** 10.1371/journal.pone.0095762

**Published:** 2014-04-21

**Authors:** Claudia A. McCarthy, Antony Vinh, Alyson A. Miller, Anders Hallberg, Mathias Alterman, Jennifer K. Callaway, Robert E. Widdop

**Affiliations:** 1 Department of Pharmacology, Monash University, Clayton, Victoria, Australia; 2 Department of Medical Sciences, RMIT University, Bundoora, Victoria, Australia; 3 Department Medicinal Chemistry, Uppsala University, Uppsala, Sweden; 4 Department of Pharmacology, University of Melbourne, Parkville, Victoria, Australia; School of Pharmacy, Texas Tech University HSC, United States of America

## Abstract

**Background:**

In this study, the neuroprotective effect of a novel nonpeptide AT2R agonist, C21, was examined in a conscious model of stroke to verify a class effect of AT2R agonists as neuroprotective agents.

**Methods and Results:**

Spontaneously hypertensive rats (SHR) were pre-treated for 5 days prior to stroke with C21 alone or in combination with the AT2R antagonist PD123319. In a separate series of experiments C21 was administered in a series of 4 doses commencing 6 hours after stroke. A focal reperfusion model of ischemia was induced in conscious SHR by administering endothelin-1 to the middle cerebral artery (MCA). Motor coordination was assessed at 1 and 3 days after stroke and post mortem analyses of infarct volumes, microglia activation and neuronal survival were performed at 72 hours post MCA occlusion. When given prior to stroke, C21 dose dependently decreased infarct volume, which is consistent with the behavioural findings illustrating an improvement in motor deficit. During the pre-treatment protocol C21 was shown to enhance microglia activation, which are likely to be evoking protection by releasing brain derived neurotrophic factor. When drug administration was delayed until 6 hours after stroke, C21 still reduced brain injury.

**Conclusion:**

These results indicate that centrally administered C21 confers neuroprotection against stroke damage. This benefit is likely to involve various mechanisms, including microglial activation of endogenous repair and enhanced cerebroperfusion. Thus, we have confirmed the neuroprotective effect of AT2R stimulation using a nonpeptide compound which highlights the clinical potential of the AT2R agonists for future development.

## Introduction

It is becoming increasingly evident that modulation of the renin-angiotensin system (RAS) can be protective against the damaging effects of stroke. Specifically, angiotensin II type 1 receptor blockers (ARBs), which are most commonly used to treat hypertension, have unique mechanisms of action that provide benefit beyond simply the control of blood pressure [1], [2]. Angiotensin II (Ang II) is the main effector hormone of the RAS that has the ability to interact with different receptor subtypes to elicit a range of physiological events. The most abundant of the Ang II receptors is the angiotensin II type 1 receptor (AT1R), responsible for most of the physiological and pathological events classically associated with Ang II [3]. The angiotensin II type 2 receptor (AT2R) is more sparsely expressed in adult tissue and therefore is often silenced by the dominant AT1R. However, in certain disease settings, including stroke, AT2R expression is up-regulated, suggesting that it may be involved in the body’s endogenous response to injury [4]–[6].

In the context of stroke, treatment with an ARB will minimise the extent of tissue damage and degree of behavioural impairment caused by experimentally induced stroke [6]–[8]. Interestingly, during AT1R blockade, the actions of the AT2R are unmasked, with indirect evidence highlighting an AT2R mediated protective influence on neurons. In fact, the stroke protection elicited by the ARBs seems to be entirely attributed to increased activation of the AT2R, as the therapeutic benefit is completely reversed when the AT2R is simultaneously blocked using the AT2R antagonist, PD123319 [4], [6], [9], [10]. Consistent with this, mice lacking the AT2R exhibit poorer stroke outcome and impaired sensitivity to the protective actions of the ARBs, again illustrating the crucial role the AT2R has in reducing stroke damage [11].

Recently, we directly examined the neuroprotective effects of the AT2R in a rat model of stroke using the agonist, CGP42112 [12], [13]. Central administration of CGP42112 produced a dose dependant reduction in infarct volume and dramatically decreased the resulting functional deficits following middle cerebral artery occlusion (MCAO) [12], [13]. Interestingly, the benefit evoked by CGP42112 was not only evident when AT2R stimulation occurred prior to stroke induction [13], but the level of protection was similar when commencement of treatment was delayed until 6 hours after ischemia [12]. These investigations were the first direct illustration of stroke protective actions of the AT2R. However, while CGP42112 provides proof of principal of AT2R mediated neuroprotection, this peptide is easily metabolised and unlikely to cross the blood brain barrier, which renders it unsuitable for use in humans.

A selective nonpeptide AT2R agonist, C21 (also known as M024), has been developed [14] which produces a similar effect to CGP42112 on blood pressure [15] and improves cardiac function following myocardial infarction [16]. The AT2R agonist C21 is similar in size and structure to the nonpeptide AT1R antagonists (ARBs) in clinic and is highly selective for the AT2R, reported to be anywhere from <4,000-fold [17] to 25,000-fold [14] selective for AT2R over AT1R. In the setting of stroke, although not specifically assessing brain injury, Gelosa *et al.,* found that oral administration of C21 delayed brain damage and extended life expectancy in stroke-prone hypertensive rats on a high sodium diet [18]. In light of our research illustrating the protective actions of the AT2R during cerebral ischemia, the aim of the current study was to examine whether the same degree of neuroprotection can be induced using the novel drug-like agonist C21, which is more clinically relevant than the previously studied peptide agonist CGP42112.

## Methods

### Surgical Procedures

#### Cannulae implantation

The guide cannula to be used during endothelin-1 (ET-1) titration was stereotaxially inserted according to methodology outlined by Sharkey *et al*,. [19]. Male spontaneously hypertensive rats (SHR) (330–350 g) were anaesthetised with ketamine (75 mg/kg; Sigma)/xylazine (10 mg/kg; Troy; i.p). A 23-gauge stainless steel guide cannula was stereotaxically implanted to sit 3 mm dorsal to the right middle cerebral artery in the piriform cortex. The stereotaxic coordinates were modified for the SHR strain of rat (0.2 mm anterior, −4.7 mm lateral, and −7 mm ventral relative to Bregma) [12], [13]. An additional cannula was implanted into the left lateral ventricle (−0.8 mm anterior, +1.5 mm lateral, and −3.2 mm ventral relative to Bregma) which was either attached to a drug filled subcutaneous minipump to allow drug to be continuously administered for 5 days prior to and 3 days after stroke (Alzet model 2 ML2) or was left exposed to allow a bolus dose of drug to be administered at 4 time points after stroke. The animals were housed individually and were allowed a 5-day recovery period prior to the induction of stroke.

### Ethics Statement

These studies were approved by a Monash University Animal Ethics Committee (Application number; MARP/2011/091).

### Treatments

#### Pre-stroke treatment

The animals were randomly allocated to one of several treatment groups: AT2R agonist C21 at doses (5, 10 or 50 ng/kg/min) (n = 8, 11, 15 respectively); C21 (50 ng/kg/min) in combination with AT2R antagonist PD123319 (36 ng/kg/min) (n = 11); PD123319 alone (36 ng/kg/min) (n = 12) and vehicle (saline) only control (n = 13). C21 has been shown to have approximately 10-fold less affinity at the AT2R compared to the classic agonist CGP42112, therefore the doses of C21 used in the concentration-response curve were derived from the dose of CGP42112 that we have previously reported to be neuroprotective^13^. Additionally, we have confirmed that PD123319 given centrally at a dose of 36 ng/kg/min will effectively block the stroke protective effects CGP42112 [12], [13], therefore the same dose of PD123319 was used in the current study.

#### Post-stroke treatment

The dose of C21 was derived from the pre-treatment series of experiments described above; the same cumulative amount of C21 at the highest dose tested (50 ng/kg/min for 8 days) was administered over 4 bolus injections performed after stroke induction. Rats were allocated to either C21 (144 µg/kg/dose) (n = 11); C21 (144 µg/kg/dose)+PD123319 (103 µg/kg/dose) (n = 6); PD123319 alone (103 µg\kg\dose) (n = 12) or vehicle (saline) control (n = 12). All drugs were administered at 4 time points after stroke: 6, 24, 48 and 72 hours post stroke and were administered via a previously implanted guide cannula directly into the left lateral ventricle using a 30-gauge injector protruding 3 mm beyond the end of the guide cannula. All drugs were dissolved in saline and infused in a volume of 3 µl over 3 minutes.

### Systolic Blood Pressure Measurement

Systolic blood pressure (SBP) was measured using a non-invasive tail-cuff blood pressure analysis system (Model MC400, Hatteras Instruments) 6 days prior to stroke and at 72 hours post-stroke. Animals were placed on a heated platform and held in a restrainer during pressure detection. 1 day prior to the initial measurement of SBP animals were habituated to the restrainer and an average of at least 4–5 determinants were calculated.

### Stroke Induction

During stroke induction, animals were placed in a clear Perspex box to allow observation. Stroke was induced in conscious animals by inserting a 30-gauge injector protruding 3 mm below the end of the previously implanted guide cannula and endothelin-1 (ET-1) (20pmol/µl in saline; AusPep) was injected at a rate of 0.2 ul every 30 seconds until the animal exhibited behavioural changes associated with the desired level of stroke. Typical behaviours that were observed were continuous contralateral and ipsilateral circling; clenching, dragging, or failure to extend the forelimb contralateral to the side of ET-1 infusion; chewing and jaw flexing and shuffling with forepaws [12], [13]. Each stroke was graded based on these pre-determined behavioural changes using a scale of 1–4, with 1 being a mild stroke and 4 being a severe stroke. Only rats with a grade 4 level of stroke, exhibiting at least 5 of the aforementioned behaviours, were used for the purpose of this investigation. Out of the 139 animals stroked in this study, 1 animal was excluded because it did not reach the appropriate level of stroke. In addition, 17 animals were excluded because they had a stroke that was greater than a level 4 stroke. All excluded animals were humanely sacrificed immediately after the final injection of ET-1. The experimenter was blinded to all treatments in these studies.

### Assessment of Functional Outcome

Motor co-ordination abnormalities were examined by assessing the animals dependence on the under-hanging wider ledge of a gradually narrowing beam as previously described [12], [13]. Naïve rats are able to traverse the central portion of the beam without using the underhanging ledges for support. Rats that have had a stroke use the ledge for support on the impaired side and take more steps on the ledge. Animals were trained to traverse the beam on the day prior to the surgical implantation of the cannalae. The ledged beam test was conducted immediately before stroke induction, at 24 hours (day 1) and ∼70 hours after stroke induction (day 3). The number of steps taken on the lower ledge (errors) by each foot was recorded and expressed as a percentage of the total number of footsteps taken and recorded as percentage error. All values were compared to pre-stroke performance, therefore each rat acted as its own control. It should be noted that the assessment of motor function at 24 hours post stroke occurred prior to the second injection of drug in the delayed treatment protocol.

### Quantification of Ischemic Damage

At 72 hours after stroke rats were re-anaesthetised with ketamine (75 mg/kg; Sigma)/xylazine (10 mg/kg; Troy) and transcardially perfused with physiologically buffered saline (0.1 M PBS; pH 7.4) at a rate of 25 ml per minute. Brains were then removed, snap frozen and sectioned for image analysis using the ballistic light method first developed by Callaway and colleagues [20].

### Immunohistochemical Staining

Neuronal expression was assessed using a neuron-specific marker, NeuN antibody (1∶500 dilution, Chemicon), which is a DNA binding protein that will bind to the nucleus of neurons. Activated microglia and brain macrophages were observed using the CD11b antibody (1∶1000 dilution, Serotec) which is commonly known as OX42 and used to visualise activated microglia [21], [22]. Frozen coronal cryostat sections (16 µm) were post-fixed in paraformaldehyde (4%). Slides were incubated overnight with either the NeuN or OX42 antibody at 4°C. Sections were then incubated at room temperature with a fluorescence labelled secondary antibody, Alexa 488 (1∶500 dilution, Invitrogen). The number of immuno-positive cells were counted within a 1 mm^2^ site for the NeuN antibody and a 0.5 mm^2^ site for the OX42-immuno staining in the infarcted region of the ipsilateral hemisphere. Ballistic light images from consecutive sections for each individual rat were used to locate the infarct and non-infarct regions in which cell counts were conducted. As a control, the number of immuno-positive cells in the identical region in the contralateral hemisphere was also counted. In addition, the number of immuno-positive cells in within the non-infarcted region of the ipsilateral hemisphere and matched region on the contralateral hemisphere was also calculated.

### Flow Cytometric Characterisation of Microglia

To identify a possible role of the AT2R in modulating microglial function, a separate group of untreated SHR were used to characterise the relationship between microglial AT2R expression and the release of the neuroprotective cytokine that has previously been associated with the AT2R [23], brain derived neurotrophic factor (BDNF). Brains were removed from sham (n = 7) and stroked (n = 9) rats at 72 hours post-stroke and enzymatically digested using collagenase type IX (125 U/mL), hyaluronidase IS (60 U/mL) and collagenase type IS (450 U/mL) dissolved in PBS containing calcium and magnesium for 30 minutes at 37°C. Digested samples were then passed through a 70 µm filter (Falcon; BR Biosciences) to yield a single-cell suspension. Leukocytes were then isolated from the cell suspension using a discontinuous percoll gradient centrifugation (30%/37%/70% isotonic percoll; GE Healthcare) for 25 minutes at room temperature at a speed of 2700 RPM. Leukocytes were then removed from the 37%/70% interface and washed with PBS and stained for surface markers for leukocytes (PE Cy7 anti-CD45 (OX1); Biolegend) and myeloid cells which include microglia and macrophages (APC anti-OX42; Biolegend). Cells were washed with staining buffer (0.5% BSA in PBS) then fixed and permeabilized and stained with primary antibodies that were specific for the AT2R (rabbit anti-AT2R; Santa Cruz Biotechnology) and BDNF (goat anti-BDNF; Lifespan Biosciences). Cells were then washed with staining buffer and stained with fluorochrome-conjugated secondary antibodies (goat-anti rabbit Alexa488 and donkey anti-goat Qdot605; Life Technologies). After a final wash cells were then analysed on a LSR II flow cytometer (BD Biosciences). Cell population analysis was conducted using FlowJo software (Version 10, Treestar). CD45^lo^+OX42+ cells were classified as microglia as previously reviewed [24].

### Vascular Reactivity

To assess the effect of C21 on cerebral artery tone, basilar arteries were excised from SHR (n = 6) and placed in cold, carbogen-bubbled (95% O_2_, 5% CO_2_) Krebs-bicarbonate solution (composition, in mmol/L; NaCl 118, KCl 4.5, MgSO_4_ 0.45, KH_2_PO_4_ 1.03, NaHCO_3_ 25, glucose 11.1, CaCl_2_ 2.5). Arteries were cut into two 5 mm rings. Ring segments were then threaded onto two wires, mounted in a Mulvany-Halpern myograph (Danish Myo Technology A/S), and resting tension was increased to either 5 mN. After an equilibration period of 30 min, rings were exposed to a high potassium physiological salt solution (KPSS) containing 122.7 mmol/L KCl (equimolar replacement of NaCl with KCl) to induce vascular contraction defined as ‘100% of KPSS’. In all rings, endothelial integrity was assessed by measuring acetylcholine (ACh, 10 µmol/L)-induced relaxations of U46619 (10 −100 nmol/L)-induced tone (50–60% of contraction to KPSS). Following wash out, rings were again contracted sub-maximally (50–60% of contraction to KPSS) with U46619. Once contractions were stable, rings were exposed to cumulative doses of either C21 (10 nmolL –1 µmol/L) or saline (time control). In some experiments, rings were pre-treated with the AT_2_ receptor antagonist PD123319 (10 µmol/L) for 30 min prior to addition of C21. At the conclusion of each experiment, rings were exposed to papaverine (100 µmol/L) to ensure maximal relaxation could be achieved.

### Statistical Analysis

Results are presented as mean±standard error of the mean (SEM). The ledged beam test, systolic blood pressure, and vasoactive responses to C21 were analysed using a 2-way RM ANOVA. The total number of microglia and proportion of cells producing BDNF was analysed using Student’s unpaired t-test. Infarct area neuronal expression, expression of the AT2R within the population of microglia and proportion of cells producing BDNF were analysed using a 1-way ANOVA. A value of P<0.05 was considered to be statistically significant; all statistical analyses were performed using GraphPad Prism (GraphPad software).

## Results

### Systolic Blood Pressure

There was no significant difference in SBP between any of the treatment groups indicating that C21 did not significantly alter SBP in either treatment protocol (Table 1).

**Table 1:** Effect of Various Treatments on Systolic Blood Pressure (SBP).

Treatment (5 days prior and 3 days after stroke)	PretreatmentSBP, mmHg	72 Hours PoststrokeSBP, mmHg
Vehicle (n = 7)	176±7	179±5
C21 (10 ng/kg/min; n = 6)	170±7	177±6
C21 (50 ng/kg/min; n = 11)	177±5	175±5
C21 (50 ng/kg/min)+PD123319 (36 ng/kg/min; n = 6)	161±8	169±6
(Delayed until 6hrs after stroke)		
Vehicle (n = 12)	174±5	176±6
C21 (144 µg/kg/dose; n = 11)	172±6	175±8
PD123319 (103 µg/kg/dose; n = 12)	177+6	179±7
C21 (144 µg/kg/dose)+PD123319 (103 µg/kg/dose; n = 6)	170±8	171±7

### Infarct Area

**Figure 1:**
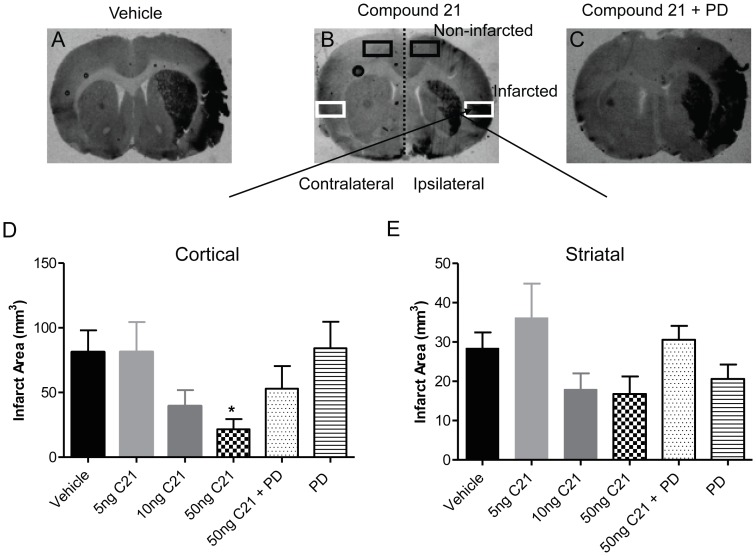
Infarct area when C21 is given as a pre-treatment. Histological sections showing typical infarcted (darker area) and non-infarcted regions from SHR that were either treated with (A) vehicle, (B) AT2R agonist C21 50 ng/kg/min or (C) C21 50 ng/kg/min+PD123319 36 ng/kg/min. Mean data±SEM for infarct volume taken 72 hours post stroke in (D) cortical and (E) striatal regions on the ipsilateral side are shown for vehicle (n = 11), C21 at 5 ng/kg/min (n = 8), 10 ng/kg/min (n = 11) and 50 ng/kg/min (n = 11), AT_2_R antagonist PD123319 alone (n = 10) and in combination with C21 50 ng/kg/min (n = 11). *P<0.05 vs. vehicle (1-way ANOVA).

**Figure 2:**
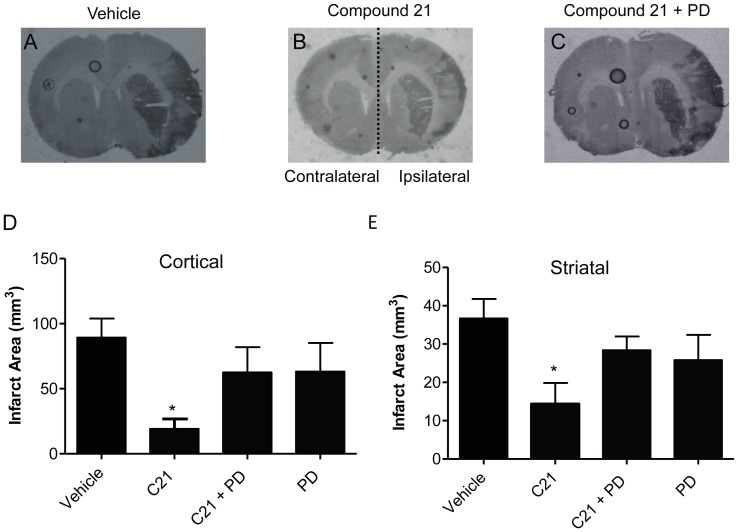
Infarct area when C21-treatment is delay until 6 hours after stroke. Histological sections showing typical infarcted (darker area) and non-infarcted regions from SHR that were either treated with (A) vehicle, (B) AT_2_R agonist C21 144 µg/kg/dose or (C) C21 144 µg/kg/dose+AT_2_R antagonist PD123319 103 µg/kg/dose. Mean data±SEM for infarct volume taken 72 hours post stroke in (D) cortical and (E) striatal regions on the ipsilateral side are shown for vehicle (n = 12), C21 (144 µg/kg/dose; n = 11), PD123319 alone (103 µg/kg/dose; n = 12) and in combination with C21 (144 µg/kg/dose; n = 6). *P<0.05 vs. vehicle (1-way ANOVA).

Intracerebroventricular (i.c.v.) administration of C21 at the highest dose (50 ng/kg/min) for 5 days prior to and 3 days post-stroke significantly reduced cortical infarct volume measured 72 hours after MCAO compared to vehicle (21.52±7.92 mm^3^; 81.14±16.93 mm^3^ respectively; P<0.05 vs. vehicle; Fig. 1). There was also a trend for C21 to decrease striatal infarct volume but this did not reach statistical significance. Furthermore, when treatment with C21 was delayed to commence 6 hours after stroke, both cortical and striatal infarct volumes were significantly reduced in the animals receiving C21 (cortical: 19.20±7.58 mm^3^; striatal: 14.36±5.50 mm^3^) compared to vehicle-treated SHR (cortical: 89.36±14.54 mm^3^; striatal: 36.69±5.08 mm^3^; P<0.05, Fig. 2). Regardless of the drug administration regime, the stroke protection afforded by C21 was attenuated by co-administration of the AT2R antagonist PD123319 while PD123319 itself had no effect when administered alone.

### Motor Deficit

**Figure 3:**
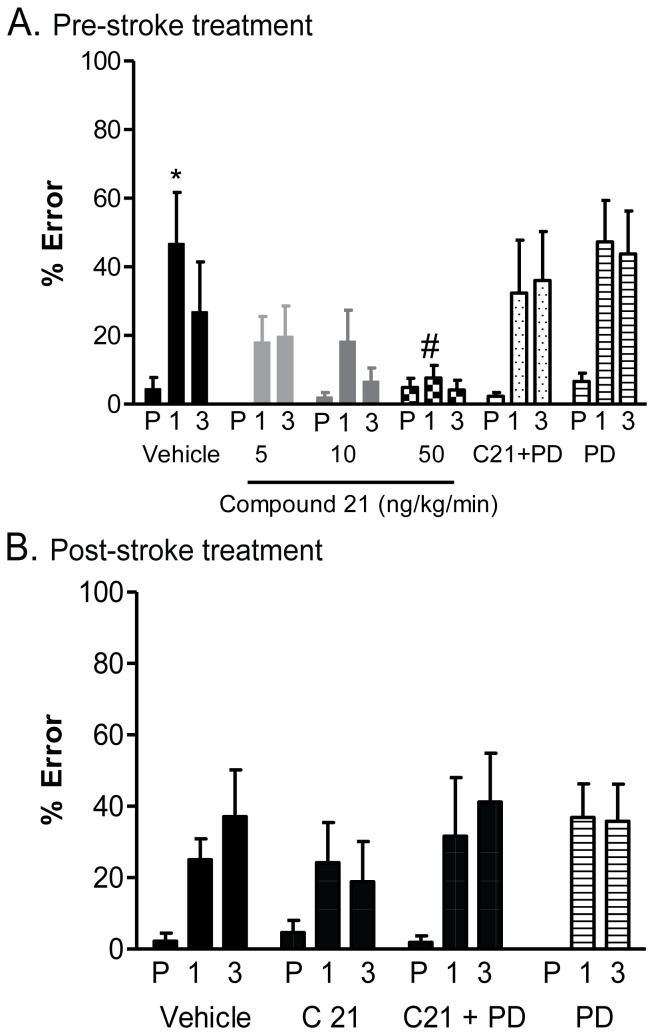
Motor deficit. Motor deficit when C21 is given either pre-stroke (A) or post-stroke (B). For pre-treatment, the effect of vehicle (saline) control (n = 9), AT_2_R agonist C21 at 5 ng/kg/min (n = 7), 10 ng/kg/min (n = 11) and 50 ng/kg/min (n = 11), AT_2_R antagonist PD123319 alone (36 ng/kg/min; n = 10) and in combination with C21 (50 ng/kg/min; n = 8) on percentage errors made on the ledged beam following stroke. For post-stroke treatment, the effect of vehicle (n = 12), AT2R agonist C21 (144 µg/kg/dose; n = 11), AT2R antagonist PD123319 alone (103 µg/kg/dose; n = 12) and in combination with C21 (144 µg/kg/dose; n = 6) on percentage errors made on the ledged beam following stroke. Ledged beam test was performed pre-stroke (P) and at 1 (1 day) and 3 (3 day) post stroke. Mean data+SEM; ^*^P<0.01 vs. vehicle pre-stroke (1-way RM ANOVA); ^#^P<0.01 vs. corresponding time in vehicle-treated group (2-way RM ANOVA).

Motor deficits were increased at both 1 and 3 days after stroke in the vehicle-treated SHR, with the severity of deficit being most pronounced 1 day after stroke in the control group in the pre-treatment series of experiments (47±15% error; Fig. 3A). The highest dose of C21 (50 ng/kg/min) administered prior to stroke significantly reduced the severity of motor deficit at 1 day after stroke (8±4% error; P<0.01 vs. corresponding time point in vehicle-treated group) when compared to matched vehicle-control. Although at 3 days post-stroke the trend for behavioural protection was still evident in these animals, this failed to reach statistical significance. Consistent with the histological data, PD123319 reversed the C21 treatment effect, while having no effect on % error when administered alone. When treatment was delayed to commence 6 hours after MCAO, there was no improvement in motor deficit in the animals receiving C21, which may be related to the fact that the degree of motor deficit induced by stroke in the vehicle-treated group was relatively modest (Fig. 3B).

### Neuronal Survival

**Figure 4:**
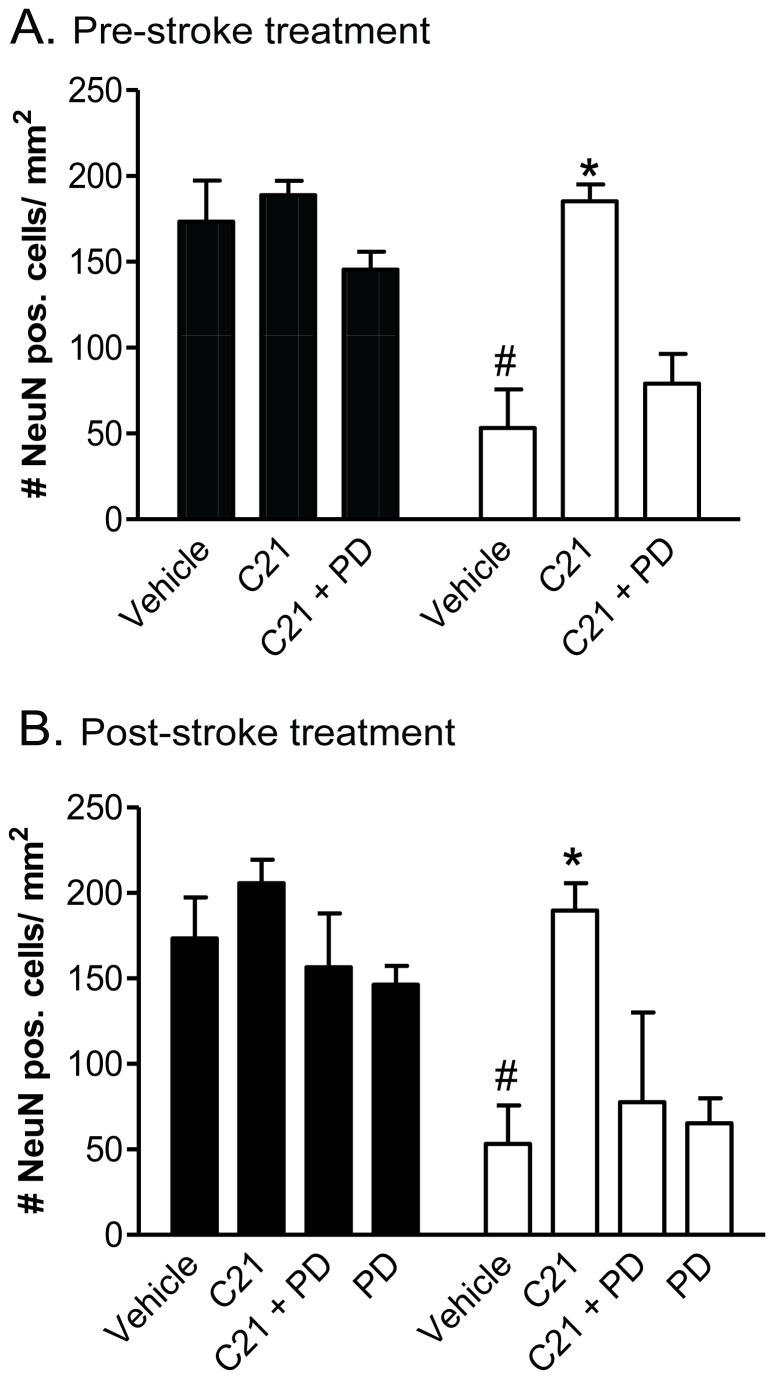
Neuronal survival. Neuronal survival when C21 is given either pre-stroke (A) or post-stroke (B). For pre-treatment, the effect of vehicle (saline; n = 7); AT_2_R agonist C21 alone (50 ng/kg/min; n = 8) and in combination with AT_2_R antagonist PD123319 (36 ng/kg/min; n = 6) on neuronal survival at 72 hours post stroke. For post-stroke treatment, the effect of vehicle (n = 8), AT2R agonist C21 (144 µg/kg/dose; n = 8), AT2R antagonist PD123319 (103 µg/kg/dose; n = 8) in combination with C21 (144 µg/kg/dose; n = 6) on neuronal survival at 72 hours post stroke. Data expressed as the number of NeuN-immunopositive cells in infarcted (open columns) and non-infarcted (filled columns) cortical regions on the ipsilateral hemisphere. Mean data±SEM. *P<0.05 vs. corresponding region in vehicle group; #P<0.05 vs. non-infarcted region within same animal (1-way ANOVA).

Following both treatment protocols, the number of neurons as detected by NeuN-immuno-positive staining was significantly lower in the infarct area (pre-treatment: 58.86±32.15; delayed treatment: 53.25±22.45 NeuN-positive cells per mm^2^) compared to a non-infarcted region within the ipsilateral hemisphere (pre-treatment; 154.90±26.44, delayed treatment; 173.50±23.97 NeuN-positive cells per mm^2^) of the vehicle-control animals. When administered prior to stroke, C21 significantly attenuated the loss of neurons in the ischemic tissue, such that the number of NeuN-positive cells in the infarcted region was comparable to the number of NeuN-positive cells in the unaffected area (153.10±17.23; 181.50±14.48 NeuN-positive cells per mm^2^ respectively. P<0.05 vs. corresponding region in vehicle; Fig. 4A). The same preservation of neuronal cells was evident when C21 was given 6 hours after stroke (non-infarcted: 188.80±23.97; infarcted: 185.40±9.64 NeuN-positive cells per mm^2^; P<0.05 vs. corresponding region in the vehicle; Fig. 4B). Furthermore, in accordance with the functional and histological data, the conservation of neurons seen in the C21-treated animals was ameliorated when PD123319 was simultaneously administered.

### Microglial Activation

**Figure 5:**
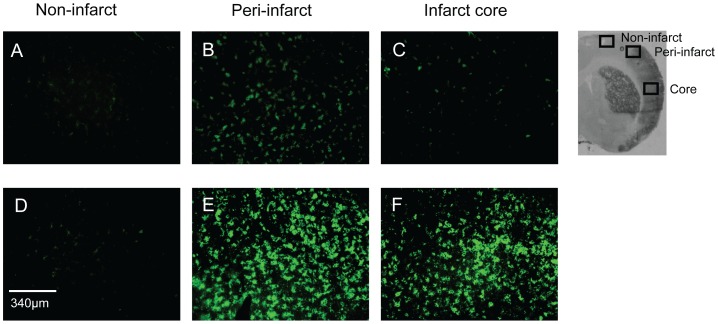
Microglial activation. Immunohistochemical sections from the cortex of the ipsilateral hemisphere from an animal receiving a pre-treatment of vehicle (A–C) or Compound 21 (50 ng/kg/min) depicting microglial activation using OX42 staining (green) in the non-infarcted tissue (A and D); the peri-infarcted tissue (B and E) or the core region of damage (C and F).

**Figure 6:**
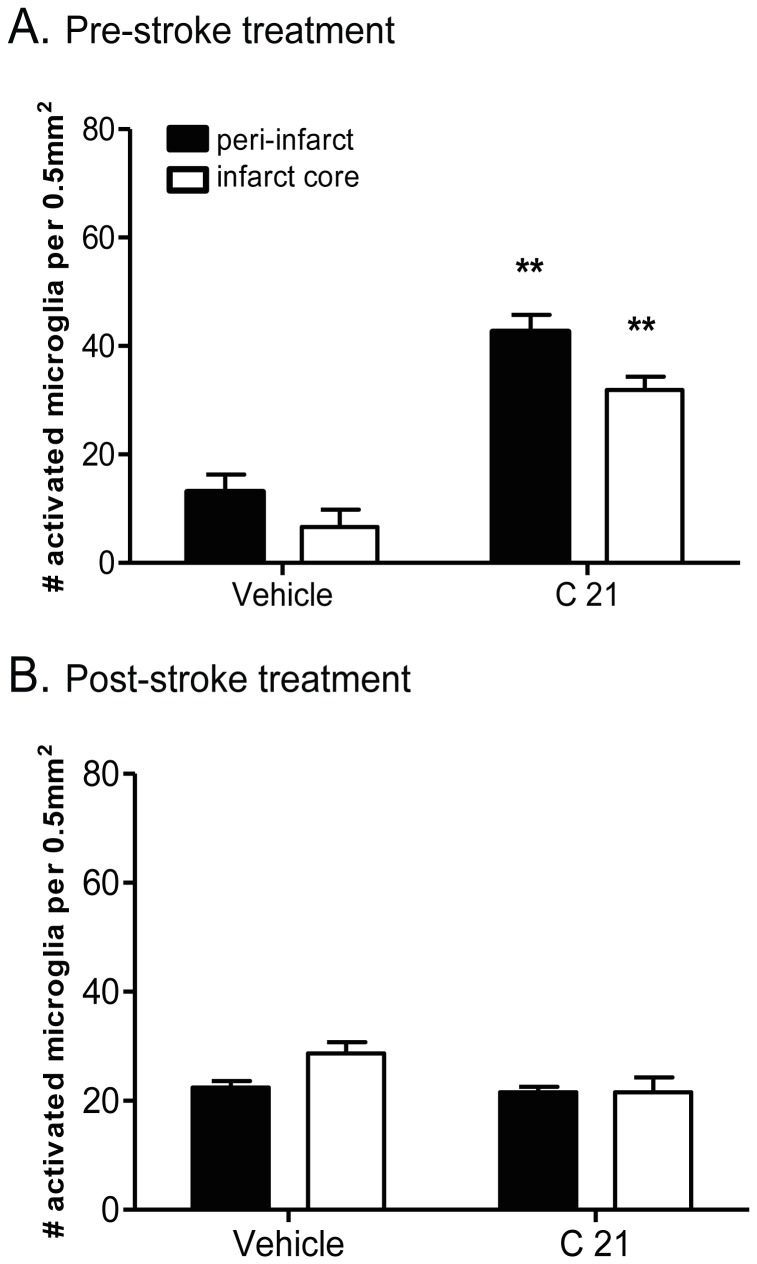
Number of activated microglial cells. Number of activated microglial cells when C21 given either pre-stroke (A) or post-stroke. For pre-treatment, the effect of vehicle (saline; n = 6) and AT_2_R agonist C21 (50 ng/kg/min; n = 5) on the number of activated microglia at 3 days after stroke. For post-stroke treatment, the effect of vehicle (saline; n = 7) and AT_2_R agonist C21 (144 µg/kg/dose; n = 7) on the number of activated microglia at 3 days after stroke. Data expressed as number of activated microglia per 0.5 mm^2^ area in non-infarcted (filled columns) and infarcted (open columns) region on the ipsilateral hemisphere. Mean data±SEM. **P<0.01 vs. corresponding region in vehicle-control (2-tailed unpaired t test).

Stroke increased the number of activated microglial, including brain macrophages, in the damaged tissue of the ipsilateral hemisphere. There was negligible microglial activation in the contralateral hemisphere (data not shown). Moreover, there was a significant increase in the number of activated microglia in the C21-pretreated animals, when compared to vehicle control, in the core region of the infarct (42.63±3.14 and 13.12±3.15 OX42-positive cells per 0.5 mm^2^ respectively; P<0.01 vs. corresponding region in vehicle; Figs 5 & 6A), as well as in the peri-infarct region (C21∶31.88±2.52 and vehicle: 6.63±3.23 OX42-positive cells per 0.5 mm^2^; P<0.01 vs. corresponding region in vehicle). However, this effect was not evident when C21 was administered 6 hours after MCAO (Fig. 6B).

### Microglial BDNF-AT2R Interaction

**Figure 7:**
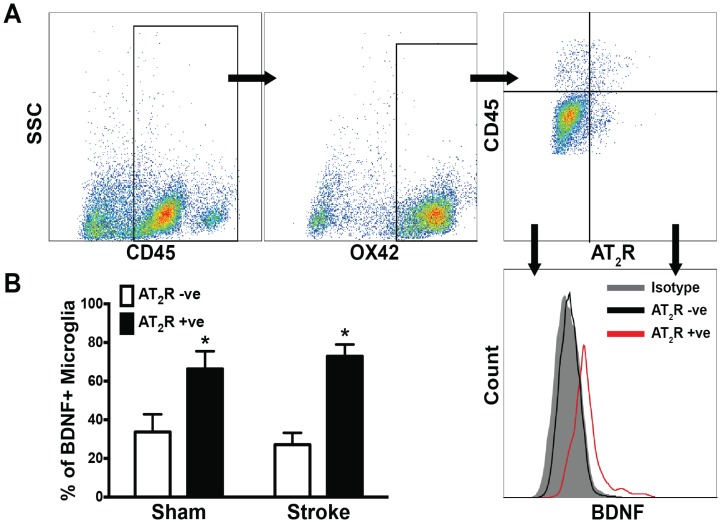
BDNF-producing microglia are predominantly AT2R-positive. Flow cytometry plots showing isolation of microglia (CD45^lo^+OX42+ positive cells) and subsequently AT2R+ positive microglia that were also positive for BDNF (A). The mean±SEM frequency of AT2R expression within the population of microglia-producing BDNF obtained from SHR brains that had 3 days previously undergone either sham or stroke surgery (B). *P<0.05 vs. AT2R negative, BDNF-producing microglia (n = 4–9).

To determine a possible involvement of the AT2R in promoting microglial release of BDNF, a neuroprotective cytokine, microglia cells were isolated 3 days after stroke and analysed using flow cytometry (Fig. 7). OX42+ cells were gated from the total CD45+ population in order to locate brain microglia within the total population of leukocytes isolated from brains. To differentiate between microglia and macrophages, CD45^lo^OX42+ cells were identified as microglia. AT2R were localised on a small population of microglia and the majority of these AT2R-positive microglia were also positive for BDNF (Fig. 7). Moreover, this phenotype was not altered by stroke since, in both sham and stroked rats, the predominant proportion (∼75%) of BDNF-producing cells were also AT2R-positive (Fig. 7).

### Vascular Reactivity

**Figure 8:**
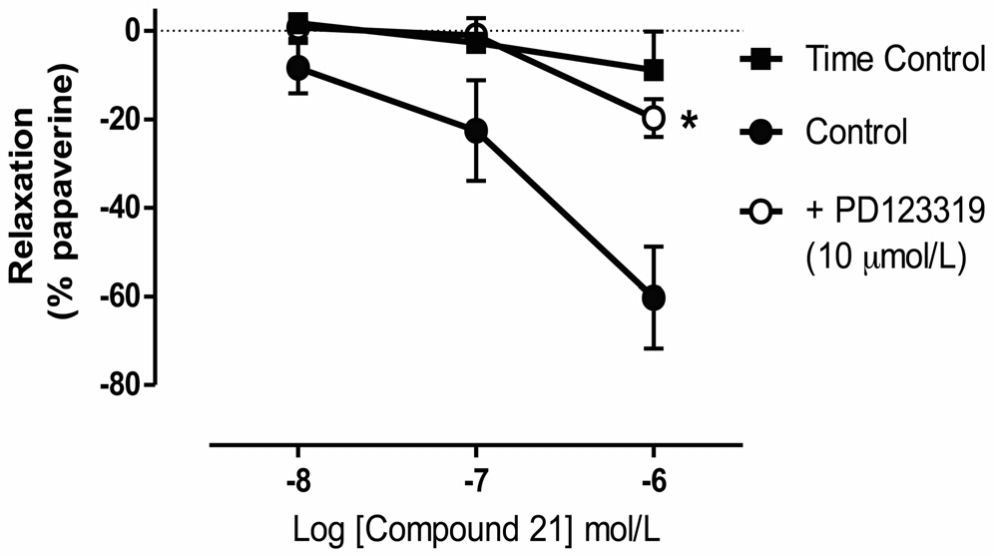
C21 elicits cerebral vasorelaxation through AT2R activation. Cumulative concentration-response curves showing relaxation responses of isolated rat basilar arteries to C21 (10 nmolL –1 µmol/L) in the absence and presence of the AT2R antagonist PD123319 (10 µmol/L). Results are expressed as percent relaxation of U46619-induced tone and are given as mean±SEM (n = 5–7). *P<0.05 vs. control, (2-way ANOVA).

The acute effect of C21 in the absence and presence of AT2R block was examined to establish whether enhanced cerebral blood flow may be a component of the C21-treatment effect. ACh (10 µmol/L) elicited similar relaxation responses in basilar arteries in C21-treated and C21+ PD123319 treated arteries (97±2%; and 94±13%, respectively) indicating a functional endothelium. Furthermore, the level of pre-contraction induced by the thromboxane A2 mimetic U46619 was similar between these two groups (C21∶50±5% max and C21+ PD123319∶44±5% max, respectively). C21 elicited concentration-dependent (10 nmolL –1 µmol/L) relaxations of basilar arteries, with a maximal effect at 1 µmol/L (R_max_: 60±12% of maximal relaxation to papaverine; Fig. 8). Moreover, relaxation responses to C21 were significantly attenuated in the presence of PD123319 (R_max_: 19±4%, P<0.05) and were comparable to those arteries not exposed to C21 (R_max_: 9±6%).

## Discussion

The major finding of this investigation is that central administration of novel non-peptide AT2R agonist, C21, protected neuronal tissue from ischaemic damage regardless of the timing of administration, such that the therapeutic benefit persisted even when treatment was delayed until 6 hours after stroke induction. The involvement of the AT2R in the stroke protection, despite relatively high local drug concentrations, was confirmed by reversal of the C21-treatment effect using the AT2R antagonist PD123319.

Using a conscious model of MCAO in SHR we showed that i.c.v. administration of C21, in a pre- or post-stroke treatment regime, reduced the severity of tissue damage independent of any changes in blood pressure. Although the gross measurement of infarct volume and the evaluation of tissue architecture both indicate that delaying treatment with C21 until after stroke was neuroprotective, there was only a modest improvement in behavioural performance. However, the control animals did not exhibit the same level of motor impairment as in the pre-treatment series of experiments, which may partly account for the lack of a statistically significant post-treatment behavioural effect. On this point, the C21-treated animals had only received a single dose of drug (25% of total dose) when the 24 hour behavioural assessment was performed, which may also account for the lack of a significant improvement in motor performance.

The evaluation of the tissue architecture using a neuronal marker (NeuN) indicates that ET-1 induced MCA occlusion caused a dramatic reduction in the number of viable neurons in the area of tissue directly affected by stroke. This loss of NeuN-positive cells was reversed by C21 during both drug administration protocols resulting in neuronal expression in ischemic tissue being similar to that seen in the unaffected cortex of the same hemisphere. While we have seen analogous AT2R-mediated preservation of neurons using CGP42112 [12], this is the first evidence that the novel agonist C21 can prevent neuronal loss to the same extent. Although a direct neuroprotective effect of AT2R stimulation was evident, an additional vasodilator role may also contribute to C21-treatment effect by augmenting cerebral blood flow and enhancing collateral circulation. It is well accepted that AT2R stimulation, including studies using C21 [15], evokes vasorelaxation [25]. In the current study, we have shown for the first time that C21 caused marked relaxation of isolated basilar arteries of SHR in a PD123319-sensitive manner, which may translate to in vivo cerebral vasodilation. However, while it is acknowledged that this in vitro analysis needs confirmation in vivo, these initial findings are in agreement with the recent report that AT2R stimulation accelerates the return of cerebral blood flow during reperfusion in the filament MCAO model of stroke [26].

Microglia are a dynamic cell population that will undergo morphological changes in response to various environmental triggers [27]. It has recently been established that microglia can polarise to exert opposing actions on surrounding neurons depending on whether an injurious or reparative phenotype is adopted [28], [29]. The conflicting views regarding the influence of microglia during injury is likely a reflection of the plasticity of these cells to polarise based on the stimuli from the local microenvironment.

In the current study, pre-treatment with C21 elevated the number of activated microglia in the core and peri-infarct regions of the ipsilateral hemisphere, which coincides with a smaller lesion area and increased neuronal survival at 3 days after stroke. In fact, augmented microglia activity appears to be a common effect of the AT2R agonists, as CGP42112-treatment produced a similar change [12]. Furthermore, there seems to be a direct relationship between the level of microglial activation and behavioural outcomes in the C21-treated animals, since both motor function and the number of OX42-positive cells were increased in the pre-treatment protocol, but not when treatment was delayed. Indeed, there is a growing body of evidence to suggest that microglia promote neuronal survival by potentiating the release of various growth factors and cytokines that accelerate wound healing, including BDNF [30], interleurkin-10 and transforming growth factor-β [31], [32]. Furthermore, the viability and growth of primary neuronal cultures, in both normal and hypoxic conditions, is enhanced when co-cultured with microglia [28].

To evaluate the relationship between the AT2R and the release of trophic cytokines, we examined microglial AT2R expression and correlated this with BDNF release. Flow cytometric analysis, in contrast to immunohistochemistry, was employed to allow the simultaneous assessment of the relative expression of the AT2R and BDNF on OX42^+^CD45^lo^ cells that were specifically identified as microglia. This methodological approach provided high resolution quantitative data that highlighted a potential functional difference between AT2R negative and AT2R positive cells. Interestingly, although the AT2R is only sparsely expressed across the total population of microglia, they are abundantly expressed on the cells that produce BDNF, and this phenotype is preserved in stroke, which implies a specific involvement of the AT2R in this cell population. Moreover, the reduction in stroke damage and concomitant increase in microglia activation in the C21-treated animals may indicate that direct activation of AT2R maintains the equilibrium between the neurotrophic and pro-inflammatory microglia. It is tempting to suggest that, when given as a pre-treatment, C21 prevented the detrimental polarising effects of stroke by enhancing the actions of the AT2R-positive-BDNF-producing microglia. In contrast, the lack of behavioural improvement and the absence of a change in the number of OX42-positive cells when C21 was given after stroke may be due to the fact that the microglia had already started to undergo polarisation at the time of drug administration. While further confirmation is required, the current results indicate that harnessing the protective influence of microglia may be a component of the beneficial effects mediated via the AT2R. Indeed, BDNF which acts to promote neuronal repair, has been indirectly associated with activation of the AT2R. Various groups have documented a PD123319-reversible increase in BDNF production [23] and BDNF receptor (TrkA) expression [33] in the neuronal tissue of candesartan-treated SHRs. More recently, C21 enhanced BDNF production in neuronal cells in vitro, which is again suggestive of a role for BDNF in AT2R signalling [34].

In conclusion, there are likely to be a number of mechanisms contributing to the neuroprotective effects seen in the present study. We have demonstrated that C21 is neuroprotective during stroke, not only when given prior to stroke, but of greater clinical significance, when treatment is delayed until 6 hours after stroke. The current findings are consistent with our previous reports which show that the AT2R agonist, CGP42112 [12], [13], protects neuronal tissue, reduces apoptosis and preserves motor function following cerebral ischemia. In fact, the analogous stroke protection afforded by structurally unrelated compounds, CGP42112 and C21, suggests that neuroprotection is a class effect of AT2R agonists. In addition, the current findings indicate that the release of trophic cytokines by microglia and enhanced vasodilatation are likely to be contributing factors. These findings firmly establish the AT2R as an important target for the development of more effective treatments for stroke.
